# Bellamy’s Surgical Anatomy

**Published:** 1874-04

**Authors:** 


The Student's Guide to Surgical Anatomy; *being a Description of the most Important Surgical Regions of the Human Body, and intended as an Introduction to Operative Surgery. By Edward Bellamy, F.R.C.S., etc.,* Teacher of Operative Surgery in the Medical School of Charing Cross Hospital, with Illustrations. Philadelphia: Henry C. Lea, 1874. 12 mo. Cloth; pp. 300. Price, $2.25.

There certainly is no dearth of works, and excellent works too, upon Surgical Anatomy, but nevertheless we believe that the neat and attractive little volume before us will meet with a cordial welcome from the profession in America. Many of the illustrations strike us as being particularly fine, for a small manual like this, and the descriptions are clear and good.

